# Validation of AI-based semen analysis by urologists in training: clinical impact after varicocelectomy

**DOI:** 10.3389/fruro.2025.1702569

**Published:** 2025-11-07

**Authors:** Giorgio Ivan Russo, Antonio Salvaggio, Maria Giovanna Asmundo

**Affiliations:** 1 Urology Section, Department of Surgery, University of Catania, Catania, Italy; 2 Urology Section, Humanitas Istituto Clinico Catanese, Catania, Italy

**Keywords:** infertility, sperm, artificial intelligence, assisted reproductive techniques, varicocelectomy

## Abstract

We conducted a prospective, single-center study (IRB 17/2025) to validate an AI-enabled, computer-assisted semen analyzer (LensHooke^®^ X1 PRO) operated by third- and fourth-year urology residents for the perioperative assessment of men undergoing loupe-assisted varicocelectomy at the University of Catania. The analyzer captured conventional and kinematic semen parameters according to the World Health Organization (WHO) 6th edition the day before and 3 months after surgery. In 42 patients (median age 31.5 years), the computer-assisted semen analysis (CASA) produced rapid, standardized readouts and showed statistically significant postoperative improvements across multiple parameters (p < 0.05). These findings support its concordance with manual analysis and underscore the value of integrating AI-based semen analysis into residency training to enhance accuracy, efficiency, and clinical decision-making in male infertility care.

## Introduction

The use of artificial intelligence (AI) is making a strong impact in the medical and surgical fields of urology (1, 2). Moreover, the potential of AI algorithms may enhance sperm selection in assisted reproductive technologies by addressing challenges such as subjectivity and inefficiency in traditional methods (3). Interpreting semen analysis is a fundamental skill for urologists in training, as it provides critical insights into male reproductive health (4). A solid understanding allows accurate diagnosis and management of male infertility, ensuring effective patient counseling and treatment planning.

Interestingly, a previous study by Agarwal et al. reported that a computer-assisted semen analyzer (CASA) demonstrated a high level of concordance with manual sperm analysis (MSA), showing strong correlations in assessing sperm concentration, total motility, and progressive motility. Notably, CASA exhibited high positive predictive values in identifying abnormal sperm parameters and demonstrated excellent inter- and intra-rater reliability. These findings suggest that this approach offers a reliable and efficient alternative to traditional manual semen analysis, potentially enhancing standardization and reducing variability in clinical assessments.

Based on these premises, the aim of this study was to validate the application of CASA by urologists in training in the clinical management of patients undergoing varicocelectomy.

## Methods

From January to March 2025 (IRB 17/2025), data were collected from patients who underwent loupe-assisted varicocelectomy at the Department of Urology, University of Catania. Each patient underwent semen analysis the day before and 3 months after surgery. Semen parameters such as pH, concentration, total and progressive motility, and morphology were determined using automated semen quality analyzers (LensHooke^®^ X1 PRO [X1 PRO]; Bonraybio, Taichung, Taiwan; software version 3.2.1, firmware f1.09). The device combines AI algorithms with autofocus optical technology to assess semen parameters.

Calibration was performed for every 50 samples. The optical configuration used a 40× objective (numerical aperture 0.65), frame rate of 60 fps, and field of view of 500 × 500 µm. The algorithm tracked sperm trajectories over ≥30 consecutive frames, discarding objects <4 µm or with non-sperm morphology. Progressive motility (PR) was defined as a velocity average path (VAP) ≥25 µm/s and straightness (STR) ≥0.80; non-progressive (NP) as motile but below those thresholds; and immotile (IM) as showing no displacement >2 µm/s. Quality-control flags were automatically raised for focus, illumination, and debris density.

Subinguinal, loupe-assisted (×3.5) varicocelectomy was performed with artery and lymphatic sparing, with optional Doppler confirmation of arterial flow and recording of veins ligated and laterality. Parameters were evaluated according to the World Health Organization (WHO) 6th-edition guidelines (5, 6). Nonconventional parameters collected included linear motility, straight motility, wobble motility, average path velocity, straight linear velocity, curvilinear velocity, amplitude of lateral head displacement, beat cross frequency, and excess residual cytoplasm.

Residents completed a structured 8 h didactic module on semen analysis principles and 10 h of supervised, hands-on sessions with the AI-CASA device. Competency was verified through two observed assessments (intra-class correlation coefficient >0.85 required). Inter-operator variability for progressive motility across residents was ICC = 0.89 (95% CI, 0.78–0.95), and intra-operator repeatability was ICC = 0.92 (95% CI, 0.85–0.96).

### Statistical analysis

Change in sperm concentration (%) from baseline to 3 months post-varicocelectomy was analyzed using a paired, within-subject design. Total motility, morphology (% normal forms), vitality (if available), and kinematic metrics (VCL, VSL, VAP, ALH, BCF, LIN, STR, WOB, PR, NP, and IM) were also evaluated.

We powered the study for the primary endpoint (progressive motility) using a paired design. Assuming a mean increase of +6 percentage points (standard deviation [SD] of differences, 12), a two-sided α = 0.05, and 80% power, the required sample size was n = 32. Allowing for 20% attrition, the target enrollment was n = 40.

For secondary endpoints and the kinematic panel, the false discovery rate (FDR) was controlled using the Benjamini–Hochberg (BH) method at q = 0.05. The primary endpoint was tested at α = 0.05 (two-sided) without adjustment.

All procedures were performed by urologists in training in their third or fourth year. Statistical analyses were conducted using Stata (Stata Statistical Software; College Station, TX: StataCorp LP), with statistical significance set at p < 0.05. Normally distributed continuous variables were reported as median (interquartile range [IQR]), and group differences were assessed using Student’s t-test or the Mann–Whitney U test, depending on the distribution (assessed by the Kolmogorov–Smirnov test).

## Results

We enrolled 42 patients with a median age of 31.5 years (IQR, 25–39). Of these, 33.3% were smokers, 7% had hypercholesterolemia, and 7% had a history of surgery for cryptorchidism or hypospadias. Most patients had grade IV (53.85%) or grade III (34.62%) left-sided varicocele according to the Sarteschi classification. The median abstinence interval was 3 days (IQR, 2–4).

**Figure 1:**
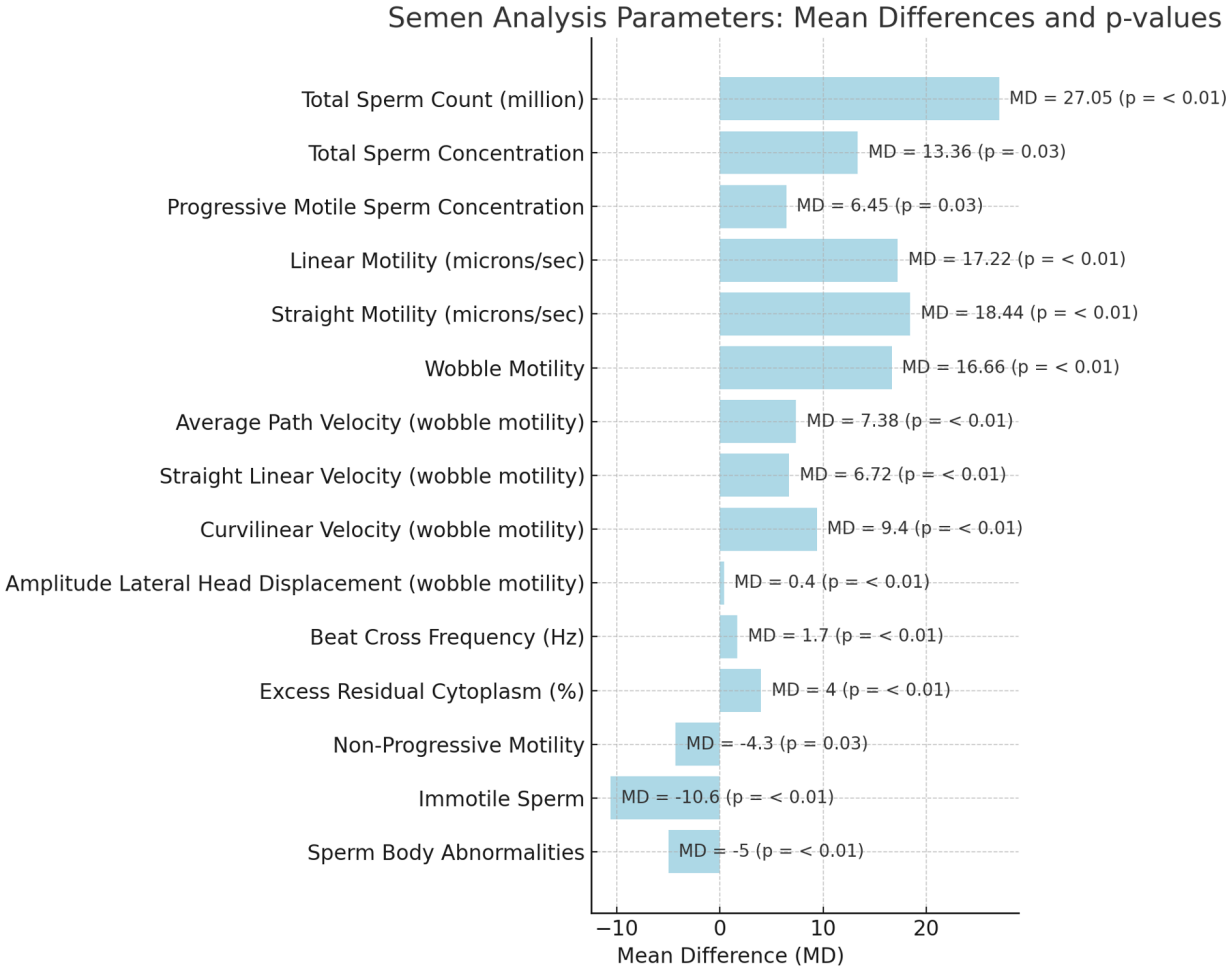
Mean differences of conventional and non-conventional sperm parameters after varicocelectomy.

**Table 1** shows baseline anatomical and semen parameters. According to CASA, sperm parameters at the 3-month follow-up compared with baseline showed statistically significant improvements in several areas (**Figure 1**).

**Table 1:** Baseline anatomical and semen parameters (preoperative).

Parameter	Median (IQR)
Age (years)	31 (25–39)
Left testis volume (mL)	10 (8–12)
Right testis volume (mL)	10 (8–12)
Semen volume (mL)	3.25 (1.6–4.5)
pH	7.8 (7.6–7.8)
Sperm concentration (million/mL)	5 (0.5–22.7)
Total motility (%)	43 (15–63)
Normal morphology (%)	4 (0–6)
BMI, kg/m^2^	24 (22.0-26.0)
Smoking, n (%)	14 (33.33)
Grade of varicocele, n (%)	
II	4 (9.5)
III	11 (26.2)
IV	27 (64.3)

Using CASA technology, based on artificial intelligence–assisted optical microscopy (AIOM) (7), results were available approximately 1 min after complete semen liquefaction, which occurred 30 min after sample collection.

## Discussion

Several computer-assisted semen analysis (CASA) systems are currently available on the market. The Sperm Class Analyzer (SCA; Microptics SL, Barcelona, Spain) assesses semen concentration and motility through image processing based on phase-contrast microscopy. The SQA-V GOLD, developed by Medical Electronic Systems (Los Angeles, CA, USA), utilizes electro-optical technology to evaluate sperm concentration and motility. Meanwhile, the IVOS and CEROS systems from Hamilton-Thorne (Beverly, MA, USA) employ integrated microscopes and cameras for advanced image-based analysis.

With continuous advancements in technology, artificial intelligence (AI)-based devices are poised to enhance both the efficiency and accuracy of semen analysis. Compared with traditional CASA systems, the X1 PRO is compact, portable, user-friendly, and demonstrates a strong correlation with manual semen analysis results. While earlier studies noted discrepancies in sperm motility assessments between manual and CASA methods, recent research highlights the X1 PRO’s capability to detect a wide range of sperm concentrations (0.1–300 million/mL) and its superior sensitivity and specificity (>90%) in identifying oligozoospermia and asthenozoospermia compared with the IVOS II.

At the core of these systems is a sophisticated use of AI to automate, standardize, and enhance the accuracy of fertility assessments. These devices rely on real-time microscopic video analysis, where AI algorithms—particularly those in the field of computer vision—identify and track sperm cells across frames. By doing so, the system can distinguish between different motility patterns, such as progressive, non-progressive, and immotile sperm, with a level of consistency and objectivity that surpasses manual analysis (3).

Although this analysis focused on short-term kinematic and hormonal outcomes, downstream clinical endpoints—such as natural conception, intrauterine insemination (IUI), or intracytoplasmic sperm injection (ICSI) success rates—are being prospectively collected in a follow-up registry. These data will clarify whether early CASA-based improvements translate into measurable fertility outcomes.

Limitations of this study should be addressed. First, we did not perform same-sample manual sperm analysis (MSA) comparisons in this cohort; therefore, agreement data were derived from prior literature rather than in-cohort validation. Second, the 3-month follow-up period could have influenced improvements in sperm parameters.

Artificial intelligence also plays a central role in quantifying various parameters such as sperm concentration, motility, and vitality. Traditional methods are often prone to human error and inter-operator variability, but the CASA approach ensures reproducibility and precision. The AI extracts detailed kinematic data, including curvilinear velocity, straight-line velocity, and beat cross frequency, providing a comprehensive profile of sperm function (8).

These systems are designed to be intuitive and accessible, often incorporating mobile connectivity and user-friendly interfaces that allow for remote analysis and reporting.

## Conclusions

The statistically significant improvements observed in both conventional and nonconventional sperm parameters following varicocelectomy underscore the clinical relevance of accurate and timely semen assessment. Most importantly, our results demonstrate that urologists in training can effectively utilize advanced CASA systems, reinforcing the need to integrate hands-on training in AI-based tools into residency programs. As technology continues to evolve, equipping future specialists with the skills to interpret and apply these innovations will be essential for advancing male reproductive health care.

## Data Availability

The raw data supporting the conclusions of this article will be made available by the authors, without undue reservation.
